# The Effects of Tgfb1 and Csf3 on Chondrogenic Differentiation of iPS Cells in 2D and 3D Culture Environment

**DOI:** 10.3390/ijms22062978

**Published:** 2021-03-15

**Authors:** Chie-Hong Wang, Chun-Hao Tsai, Tsung-Li Lin, Shih-Ping Liu

**Affiliations:** 1 Cell Therapy Center, China Medical University Hospital, Taichung 404332, Taiwan; t35383@mail.cmuh.org.tw; 2 Department of Orthopedics, China Medical University Hospital, Taichung 404332, Taiwan; ritsai8615@gmail.com; 3 Department of Sports Medicine, College of Health Care, China Medical University, Taichung 406040, Taiwan; 4 Graduate Institute of Biomedical Sciences, China Medical University, Taichung 406040, Taiwan; 5 Ph.D. Program for Aging, College of Medicine, China Medical University, Taichung 406040, Taiwan; 6 Center for Translational Medicine, China Medical University Hospital, Taichung 404332, Taiwan; 7 Department of Social Work, Asia University, Taichung 41354, Taiwan

**Keywords:** iPS cells, chondrocyte, Tgfb1, Csf3, 3D environment

## Abstract

Mesenchymal stem (MS) cells, embryonic stem (ES) cells, and induced pluripotent stem (iPS) cells are known for their ability to differentiate into different lineages, including chondrocytes in culture. However, the existing protocol for chondrocyte differentiation is time consuming and labor intensive. To improve and simplify the differentiation strategy, we have explored the effects of interactions between growth factors (transforming growth factor β1 (Tgfb1) and colony stimulating factor 3 (Csf3), and culture environments (2D monolayer and 3D nanofiber scaffold) on chondrogenic differentiation. For this, we have examined cell morphologies, proliferation rates, viability, and gene expression profiles, and characterized the cartilaginous matrix formed in the chondrogenic cultures under different treatment regimens. Our data show that 3D cultures support higher proliferation rate than the 2D cultures. Tgfb1 promotes cell proliferation and viability in both types of culture, whereas Csf3 shows positive effects only in 3D cultures. Interestingly, our results indicate that the combined treatments of Tgfb1 and Csf3 do not affect cell proliferation and viability. The expression of cartilaginous matrix in different treatment groups indicates the presence of chondrocytes. We found that, at the end of differentiation stage 1, pluripotent markers were downregulated, while the mesodermal marker was upregulated. However, the expression of chondrogenic markers (col2a1 and aggrecan) was upregulated only in the 3D cultures. Here, we report an efficient, scalable, and convenient protocol for chondrogenic differentiation of iPS cells, and our data suggest that a 3D culture environment, combined with tgfb1 and csf3 treatment, promotes the chondrogenic differentiation.

## 1. Introduction

Articular cartilage (AC) is composed of chondrocytes that are essential for matrix synthesis and maintenance [1]. When the traumatic or pathological damages to AC do not heal spontaneously, osteoarthritis (OA) sets in. In the elderly, OA is the most common disease [2,3]. Current treatments are limited to symptomatic relief through surgical procedures and medicines [4], as cartilage injury and OA do not have effective therapeutic solutions.

Stem cell-based therapies are a developing approach with great potential to treat cartilage injury and OA. Embryonic stem (ES) cells and induced pluripotent stem (iPS) cells are the most versatile among different stem cells, and can differentiate into all three germ layers, which is an important criterion for producing cells suitable for therapeutic purposes. The concept of cartilage regeneration with stem cells is established by using mesenchymal stem cells (MSCs), embryonic stem cells, induced pluripotent stem cells, and chondrogenic stem/progenitor cells [5]. However, there are still many obstacles to overcome. For example, the transplantation of MSCs tends to differentiate into tissues composed of hypertrophic chondrocytes, chondrocytes, and fibrous tissues. While there are reports of the use of ES cells as therapeutic agents in animal models [6,7,8], at least two major challenges, i.e., post-transplantation immune rejection and ethical issues, must be effectively addressed before their use on humans. Pluripotency of the ES cells endows them with an exciting potential for use in human tissue replacement and repair [9]. The application of chondrogenic stem/progenitor cells is compromised by a lacking of well-defined markers [10]. iPS cells, generated from somatic cells using Yamanaka factors, Oct4, Sox2, c-Myc, and Klf4 [11], are similar to ES cells in their proliferation, morphology, gene expression, surface antigens, epigenetic status of pluripotent cell-specific genes, and telomerase activity. The use of a patient’s own somatic cells to generate therapeutic iPS cells rules out immune rejection, and it also eliminates the use of human embryos for stem cell production. An added benefit is that patient-specific iPS cells can be employed for drug and regenerative medical research. Although the high cost of the clinical application of iPS cells is expected, the generation of HLA-homozygous iPS cells can overcome this issue [12].

Several protocols for chondrogenic differentiation of iPS and ES cells have been developed; however, these are time-consuming and labor intensive [13,14]. For example, the formation of embryoid bodies (EB) takes more than 3 weeks, and the micro-mass, cell aggregates, and cell pellet culture need to be carefully handled to avoid disturbing the cell clumps. Moreover, a 3D cell culture system has been developed for chondrocytes to overcome their de-differentiation in 2D monolayer culture during in vitro expansion [15]. To examine the biological and biomechanical changes during the expansion and/or differentiation of chondrocytes, several 3D scaffolds have been used so far [16]. Even though the use of 3D hydrogel scaffolds for chondrocyte differentiation has been well-established [17], it is still not very convenient or easy to use for encapsulation of cells in the hydrogel.

Here, we describe a simple and effective chondrogenic differentiation protocol using a nanofiber scaffold plate, serum-free culture, and mouse iPS cell clone (iPS-OSH cells) that was reported in our previous study [18]. Using our model, mouse chondrocytes can be generated within 10 days. We also assessed the effects of Tgfb1 and Csf3 on chondrogenic differentiation of iPS cells, as a previous report [19] has shown that both these molecules promote the proliferation and migration of chondrocytes.

## 2. Results

### 2.1. The Strategy to Induce Chondrogenic Differentiation in iPS Cells 

**Figure 1:**
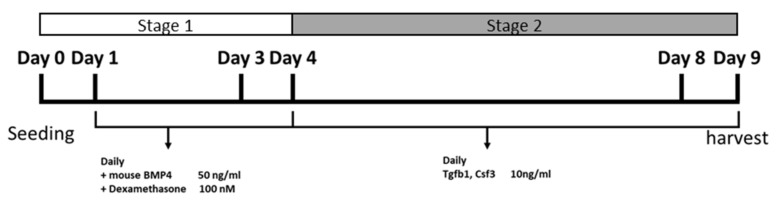
Overview of the two stage chondrogenic differentiation protocol. In the first stage, through Bmp4 and dexamethasone stimulation, the iPS cells were first differentiated into mesodermal cells. In the second stage, these cells were further treated with Tgfb1 and/or Csf3 for 5 days, to induce chondrogenic differentiation. At the end of each stage, samples were collected for examination.

In this study, we differentiated iPS cells into chondrocytes in a 2D monolayer cell culture, and nanofiber plates that mimic the 3D culture environment. The chondrogenic differentiation was divided into two stages (Figure 1). Briefly, at stage 1, iPS cells were differentiated into mesodermal cells through the stimulation with Bmp4 and dexamethasone in standard chondrogenic medium, and, at stage 2, these cells were differentiated into chondrocytes in a standard chondrogenic medium supplemented with Tgfb1 and/or Csf3. Based on the growth factors included, the chondrogenic cultures were divided into four subgroups, i.e., control group (mock), Tgfb1 group (T), Csf3 group (C), and Tgfb1-Csf3 co-treatment group (TC). 

### 2.2. Morphology of iPS Cultures at Different Stages of Chondrogenic Differentiation 

**Figure 2:**
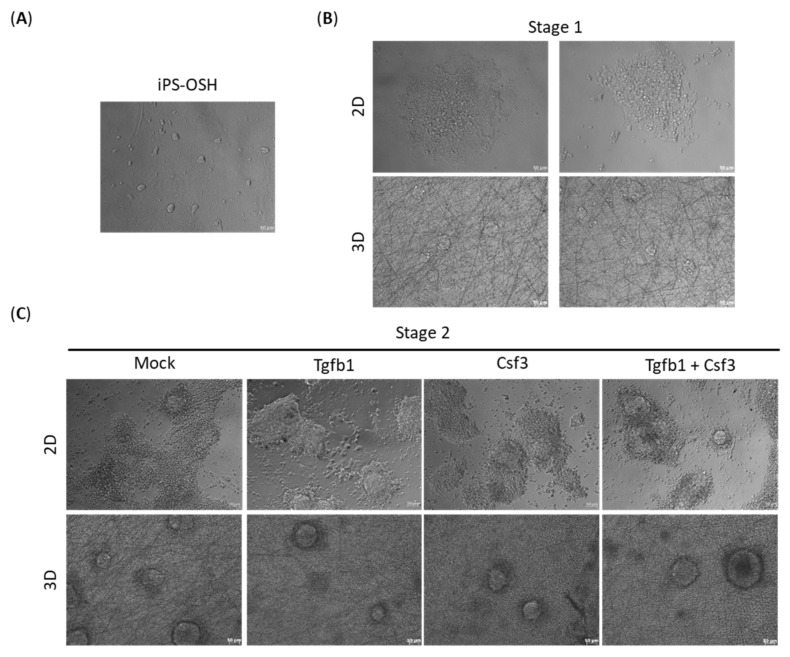

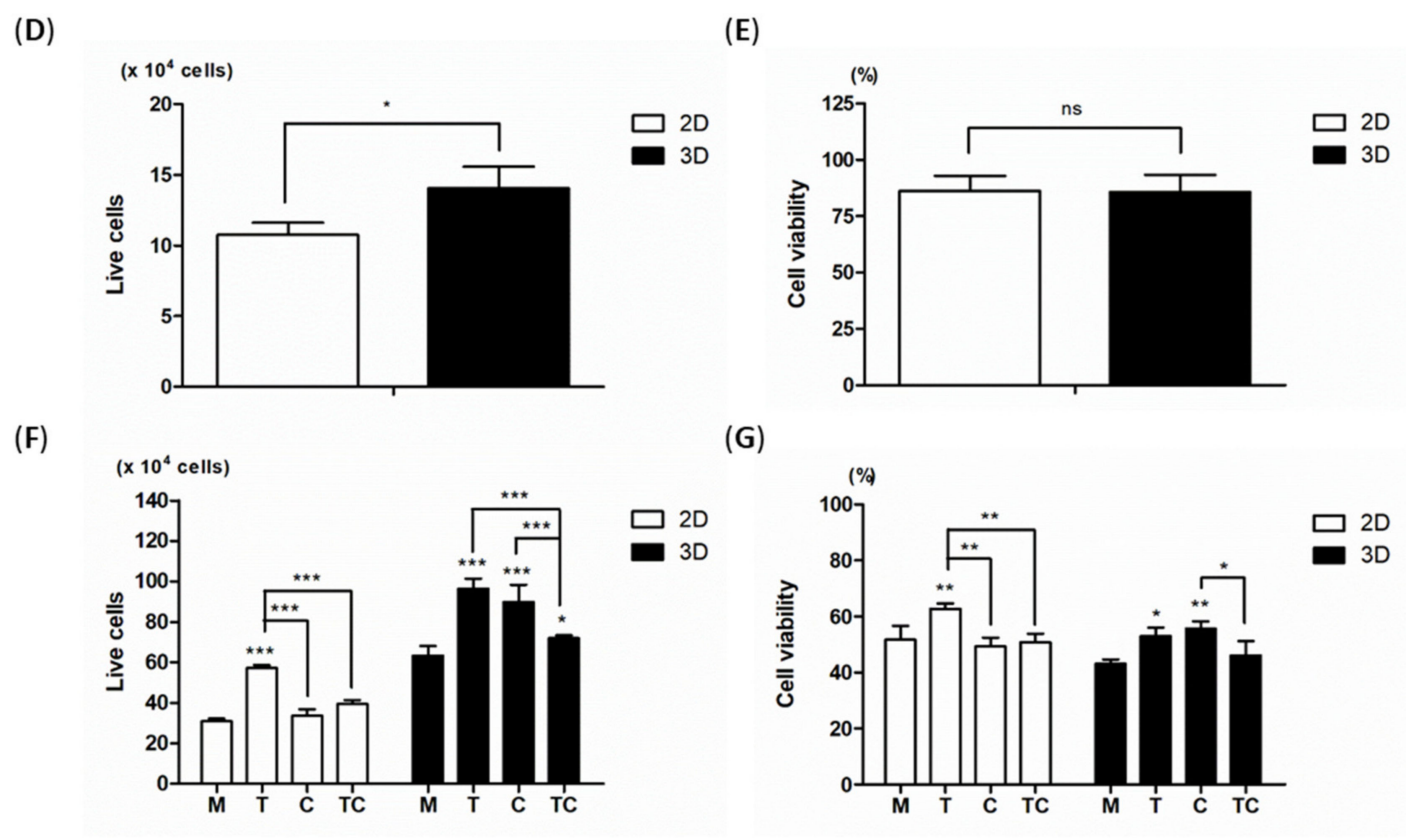
Effects of growth factors and culture environments on cell proliferation rates and viability during different stages of chondrogenic differentiation. (**A**) Morphology of iPS-OSH colonies grown on feeder cells. (**B**) Morphologies of chondrogenic cell cultures grown in 2D and 3D cultures at the end of stage 1, and (**C**) at the end of stage 2. (**D**) Effects of culture environments on cell proliferation, and (**E**) cell viability, at the end of stage 1. (**F**) The effects of growth factors and culture conditions on cell proliferation, and (**G**) cell viability, at the end of stage 2. The number of viable cells and cell viability were determined using trypan blue exclusion assay (M: mock; T: Tgfb1; C: Csf3; TC: Tgfb1 and Csf3). Bars represent mean and SD. Difference between groups were evaluated by two-tailed Student’s t test or one-way analysis of variance with Newman-Keuls Multiple Comparison Test (* *p* = 0.01–0.05; ** *p* = 0.001–0.01; *** *p* < 0.001, ns: no statistically significant difference).

The iPS cells were cultured on a feeder layer of mitomycin C-treated mouse embryonic fibroblasts (Figure 2A). Single cell suspension of iPS cells was seeded on gelatin-coated regular cell culture plates and nanofiber plates in standard chondrogenic medium, and treated with Bmp4 and dexamethasone. The iPS cells cultured on a regular 2D-plate showed a multilayered center surrounded by a monolayer of differentiated cells after 3 days of incubation, whereas those cultured on a 3D nanofiber scaffold formed small spherical multilayered colonies (Figure 2B). In the next stage, the cells were treated with standard chondrogenic medium supplemented with Tgfb1 and/or Csf3 for 5 days. At the end of this stage, cultures in all 2D subgroups showed multilayered cell clumps attached firmly to the cultureware, and a poorly adhering peripheral monolayer of cells (Figure 2C, upper panel). All 3D subgroups revealed spherical aggregates of cells with transparent-to-dark centers which were stably attached to the nanofiber plates (Figure 2C, lower panel).

### 2.3. Proliferation Rates and Viability of Cell in iPS Cultures at Different Stages of the Protocol

We examined the influence of growth factors and culture environments on cell proliferation and viability at the end of each stage of differentiation. Our data showed that the iPS cells growing in 3D cultures had higher proliferation rates than those in 2D cultures (Figure 2D), while there was no difference between the two in cell viability at the end of stage 1 (Figure 2E). However, at the end of stage 2, all the subgroups of the 3D cultures showed significantly enhanced cell proliferation as compared to their corresponding subgroups of the 2D cultures (Figure 2F). In addition, the T subgroups showed the highest cell proliferation rates in both 2D and 3D cultures. However, our results showed that treatment with Csf3 nullified the cell growth-promoting effect of Tgfb1 in both environments (Figure 2F,G). Interestingly, treatment with Csf3 alone enhanced cell proliferation, albeit in the 3D cultures only. Our results indicate that Tgfb1 enhances cell proliferation and viability in both environments, while Csf3 is effective only in the 3D environment at the end of stage 2 (Figure 2G).

### 2.4. The Accumulation of Sulfated Glycosaminoglycans

**Figure 3:**
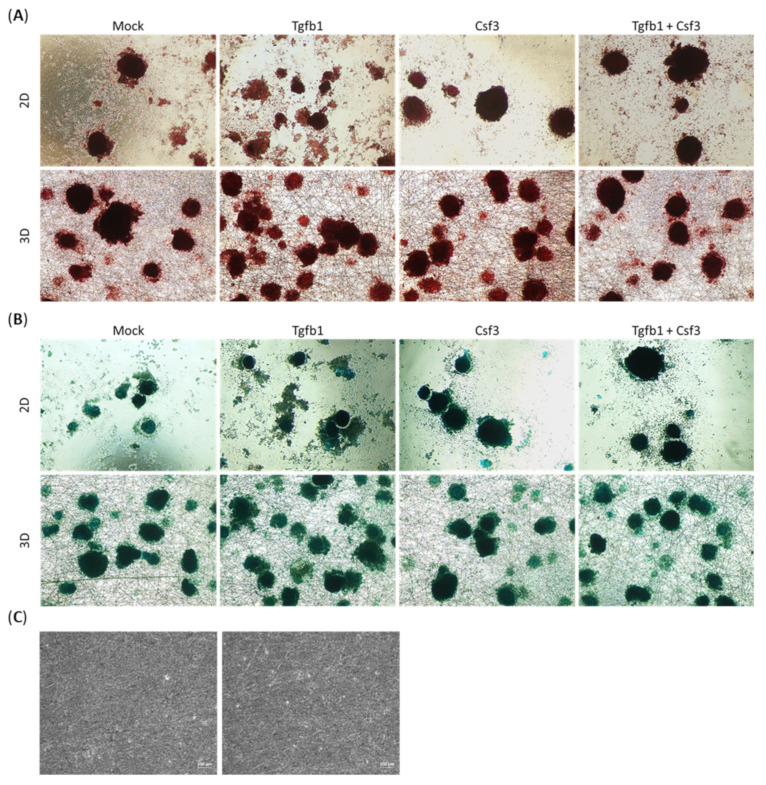
Representative images of safranin O and alcian blue stained chondrogenic aggregates at the end of stage 2. (**A**) Safranin O staining to detect the expression of acidic proteoglycan. (**B**) Alcian blue staining to localize the expression of acid mucosubstances and acetic mucin. (**C**) The photos of 3D scaffold which stained with safrain O (left panel) and alcian blue (right panel). Images were captured under phase contrast microscope, at 100× magnification.

Numbers of chondrogenic cultures in the 2D environment were clearly less than those in the 3D environment at the end of the differentiation period (Figure 3A,B), regardless of the growth factors. Staining with safranin O and alcian blue detected glycosaminoglycans (GAGs) and cartilaginous matrix in the chondrogenic cultures in both environments. The multilayered cell clumps or spheres were strongly stained, whereas the peripheral monolayers showed weak staining in all subgroups. Treatments with Tgfb1, CSf3, both separately and together, increased the number of deep-stained cell clumps and spheres in both environments, which indicated their positive role on promoting chondrogenesis. Notably, the 3D scaffold was not stained by safranin O and alcian blue (Figure 3C).

### 2.5. Gene Expression Analysis

**Figure 4:**
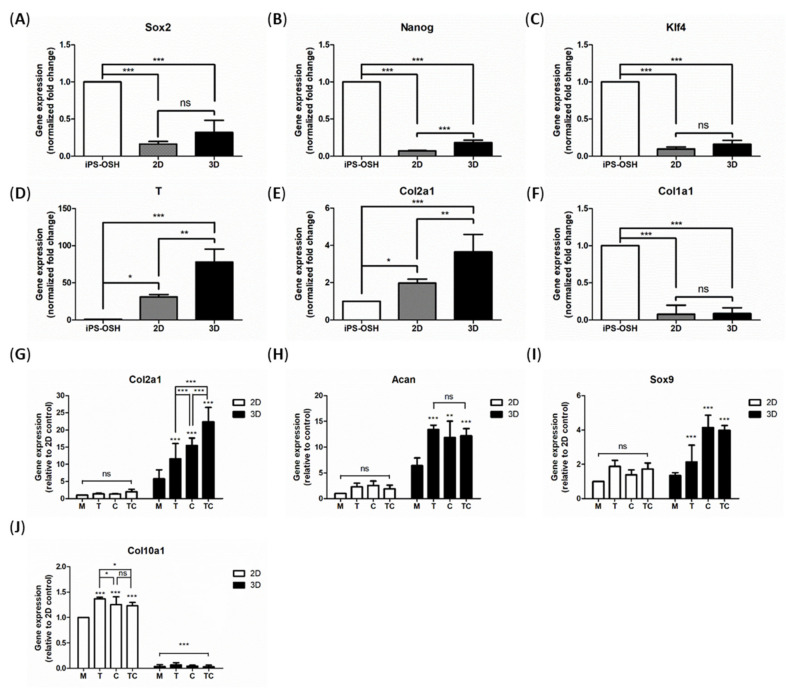
Gene expression profiles. The RNA samples of chondrogenic cultures at the end of each stages were extracted by Trizol reagent according to the manufacturer protocol. The RNA samples collected at the end of stage 1 were used to examine the expression of stem cell markers: Sox2 (**A**), Nanog (**B**) and Klf4 (**C**); mesodermal marker T-box transcription factor T (abbreviation: T) (**D**); chondrogenic marker: Col2a1 (**E**), and osteogenic differentiation marker: Col1a1 (**F**) in different groups. The RNA samples collected at the end of stage 2 were used to examine the expression of Col2a1 (**G**), Aggrecan (Acan) (**H**), Sox9 (**I**), and Col10a1 (**J**) among 2D and 3D cultures of chondrogenic cells, with and without growth factors. Data are shown as the relative fold-change compared to parental iPS-OSH cells or 2D control group, from at least three independent experiments. The expression of Gapdh was detected as the reference gene. Bars represent mean and SD. Difference between groups was evaluated by one-way analysis of variance with Newman–Keuls Multiple Comparison Test (* *p* = 0.01–0.05; ** *p* = 0.001–0.01, *** *p* < 0.001, ns: no statistically significant difference).

The total RNA extracted from chondrogenic cultures in both environments at each stage was examined by real-time PCR (Figure 4), using the primer sets listed in Table 1. The gene expression levels were normalized to Gapdh. We found that the expression levels of pluripotency genes, Sox2, Nanog, and Klf4 (Figure 4A–C, respectively), were downregulated at the end of stage 1, whereas the expression of the mesodermal marker T-box transcription factor T (T) was upregulated in both environments; however, the latter’s expression was higher in the 3D cultures than in the 2D cultures (Figure 4D). As shown in Figure 4F, the expression level of the osteogenic differentiation marker, Col1a1, was reduced in both cultures at the end of stage 1, whereas the expression of the chondrogenic marker, Col2a1, was enhanced, especially in the 3D cultures (Figure 4E), even at the end of stage 2. To our surprise, both the growth factors showed no effects in the 2D cultures (Figure 4G, left panel). However, all subgroups of the 3D chondrogenic cultures showed dramatically increased expression of Col2a1 (Figure 4G, right panel), and this was promoted by Tgfb1 and CSf3 separately, and in combination; the combined treatment resulted in the highest expression. These treatments also promoted the expression of Acan, the major proteoglycan of cartilage, among all the subgroups of the 3D cultures, but none of the 2D cultures (Figure 4H). However, among the different subgroups of 3D cultures, there was no difference (Figure 4H, right panel). Another critical regulator of chondrogenesis is Sox9 which has been validated as a master regulatory gene [20]. We found that the expression of Sox9 was enhanced in the 3D cultures as its expression was only mildly upregulated in the 2D cultures (Figure 4I). Furthermore, the expression of Col10a1, a marker of hypertrophic chondrocytes and osteogenesis, was dramatically downregulated in the 3D cultures but not in the 2D cultures (Figure 4J).

## 3. Discussion

**Figure 5:**
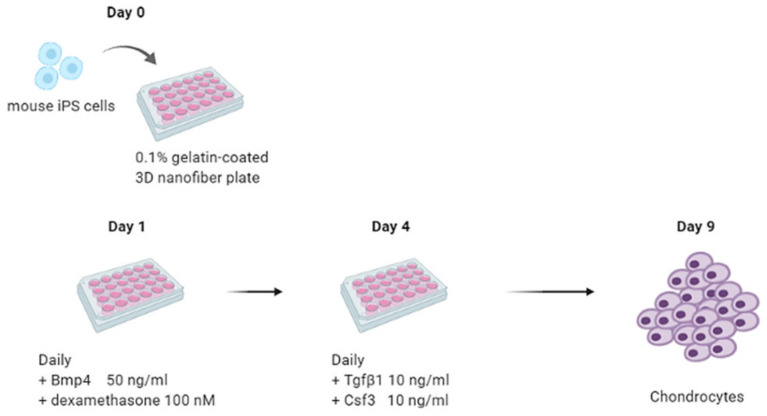
Schematic of our 3D nanofiber chondrogenic differentiation strategy. Single cell suspension of mouse iPS cells was seeded onto 0.1% gelatin pre-coated 3D nanofiber 24-well plate in standard chondrogenic medium at a density of 5 × 10^3^ cells/cm^2^ (day 0). The next day, spent medium was aspirated and standard chondrogenic medium supplied with Bmp4 (50 ng/mL) and dexamethasone (100 nM) was added (day 1). The standard chondrogenic medium supplied with Bmp4 and dexamethasone was refreshed every day. At day 4, the spent medium was replaced with standard chondrogenic medium supplied with Tgfb1 (10 ng/mL) and Csf3 (10 ng/mL). The medium was refreshed every day until day 9. The chondrogenic cultures could be collected at day 9 or for further application.

Here, we report an in vitro differentiation protocol for chondrocytes that is easy-to-handle, highly efficient, and less time consuming (Figure 5). We have evaluated the effects of different growth factors and culture environments on chondrogenesis. Most studies and commercial products conduct chondrogenic differentiation with embryoid body (EB) formation or 3D-like micromass culture [16,21,22], using differentiation protocols that take an average of 30 to 45 days, which increases the chances of contamination. In our preliminary tests, we found that it was difficult to establish the micromass culture on the nanofiber scaffold plates, as the culture surface of the 3D nanofiber plates is not absolutely flat. These droplets are flattened out by gravity when the surface tension is not able to pull them into a spherical shape. Therefore, we seeded single cell suspension onto gelatin-coated plates. We found that these cells grew into multilayered cell clumps or spheres according to the culture environments. Although iPS cells can differentiate into chondrocytes in both culture systems, the number of viable cells in the 3D chondrogenic cultures was significantly higher than those in the 2D cultures. This indicated that the 3D environment not only helps chondrogenesis, but also promotes cell expansion.

Our study optimized the chondrogenic differentiation protocol in several aspects. First, the efficiency of chondrogenesis, including cell density [23], is normally affected by several factors, including basal culture medium and the concentration of fetal bovine serum [24]. In our protocol, chondrogenesis can be induced at a cell density of 5000 cells/cm^2^, which is considerably less than the number of cells needed for EB or micromass culture protocols. Secondly, previous studies showed that activin A was required for mesodermal differentiation under serum-free conditions. Bmp4 also promotes the expression of mesodermal markers at a low dose (10 ng/mL), while at a higher dose (20 ng/mL), it inhibits the expression of the myogenic marker Myf5 [23,25]. In our study, we found that 72 h treatment with Bmp4 (50 ng/mL) and dexamethasone (100 nM) together, induced mesodermal cells, and extending this treatment to 5 days resulted in chondrogenic differentiation. This was confirmed by the presence of glycosaminoglycans and cartilaginous matrix in the cultures, and by the expression of chondrogenic markers Col2a1, Acan and Sox9. This treatment also reduced the expression of the hypertrophic marker, Col10a1. Finally, we also optimized the formulation of differentiation medium to avoid serum-induced cell apoptosis during chondrogenesis [26]. Our standard chondrogenic medium contained DMEM/high glucose, Knockout serum replacement (KSR), B27 supplement, ITS+ premix, L-proline, L-ascorbic acid 2-phosphate, GlutaMax, beta-mercaptoethanol, NEAA, and antibiotics. The DMEM/high glucose medium reduced cell death in the center of cell clumps and spheres. KSR, B27 supplement, ITS+ premix, and GlutaMax were used to promote differentiation and cell survival in the serum-free medium. In addition, 0.1% gelatin was used to coat the culture plates, as gelatin is reported to promote chondrogenesis [27]. Taken together, these results show that our protocol can be employed for the rapid screening of drugs that target chondrogenesis.

Our data indicated that effects of Tgfb1 and Csf3 on chondrogenesis differ in 2D and 3D cultures. For instance, we found that Tfgb1 and/or CSf3 did not favor chondrogenesis in 2D cultures, whereas in 3D cultures, they promoted chondrogenesis. This suggests that the interactions between growth factors and culture environments play an important role in chondrogenesis. Previously reported promotion of chondrogenic differentiation by Tgfb1, and the acceleration of proliferation of chondrocytes by Csf3, were dramatically enhanced in the 3D environment. In this study, we found that Csf3 improved chondrogenesis, and promoted cell proliferation in the 3D cultures. In all, our data indicated that Csf3 can induce iPS cells to differentiate into chondrocytes.

Although several studies have reported that chondrogenesis can be induced in 2D monolayer cultures [23,28,29], the gene expression profiles of these chondrogenic cultures may be altered by monolayer expansion [16]. For example, the increasing expression levels of Col1a1 and Col10a1 was found in monolayer culture across passage. At the same time, the expression of Col2a1, Acan and Sox9 decreases across passage in monolayer culture. This phenomenon indicated the de-differentiation of chondrocytes in prolonged 2D monolayer culture. A previous report showed that the formation of EBs and the amplification of outgrowth cells or mesenchymal stem cells (MSCs) from them, was required for chondrogenesis in the monolayer cultures [22]. However, this regular protocol is time consuming and labor intensive (e.g., about 10 days for the formation and expansion of EB and EB outgrowth cells; two to three weeks for the chondrogenesis, repsectively). In the present study, we have proposed a simplified 3D culture protocol for chondrogenesis and to evaluate the effects of different growth factors on the process. We started our chondrogenic culture with a single-cell suspension of iPS cells and 3D nanofiber plates. Although our protocol was formulated for mouse iPS cells, it can be adapted for human iPS or embryonic stem cells using different sets of growth factors, such as WNT3A, Activin-A, and FGF2 [29]. On the whole, we have established an easier and quicker protocol for in vitro chondrogenic differentiation, with which a drug screen platform can be easily established.

## 4. Materials and Methods

### 4.1. Culture of Mouse Embryonic Fibroblast Cells 

Primary mouse embryonic fibroblasts (MEFs) were isolated from 13.5-day embryos of pregnant C57BL/6JNarl mice. Briefly, the embryos were retrieved by Cesarean section. The internal organs, limbs, tail and brain were removed from the embryos. After this, the embryos were minced with scissors into fine tissue fragments and digested with trypsin-EDTA. The released MEFs were cultured in DMEM (GIBCO BRL, MA, USA) with 10% heat-inactivated FBS (Cytiva, MA, USA), penicillin (100 U/mL), streptomycin (100 ug/mL), non-essential amino acids (0.1 mM) and L-glutamine (2 mM) in a humidified 37 °C incubator with 5% CO_2_. Experimental protocols were approved by the Institutional Animal Care and Use Committee of the China Medical University (CMUIACUC-2018-308).

### 4.2. Culture of the iPS-OSH Cells

The iPS-OSH cells were established in our previous study [18]. Briefly, the mouse Oct4 and Sox2 were transient over-expressed in MEFs, and these transfected MEFs were cultured under hypoxia condition for 24 h. The ES cell-like clones were collected on day 21 and cultured on feeder cells. The MEFs pre-treated with mitomycin C were used as the feeder layers, and the iPS-OSH cells were seeded onto the feeder layers. After seeding, the iPS cells culture medium (DMEM with 15% FBS, non-essential amino acids (0.1 mM), L-glutamine (2 mM), beta-mercaptoethanol (0.1 mM) and LIF (10^3^ unit/mL)) was used to culture the iPS cells. 

### 4.3. Differentiation of the iPS-OSH Cells into Chondrocytes 

Feeder cells were removed by seeding single cell suspension onto 0.1% gelatin pre-coated tissue culture plastic for 45 min. The suspension cells were collected and seeded onto 0.1% gelatin pre-coated regular or 3D nanofiber plate (Sigma-Aldrich, St. Louis, MO, USA) in standard chondrogenic medium (DMEM/High glucose, 2% Knockout serum replacement (KSR), 1X B27 supplement, 1X ITS+ premix, 40 μg/mL of L-proline, 50 μg/mL of L-ascorbic acid 2-phosphate, 1X GlutaMax, 0.1 mM beta-mercaptoethanol, NEAA and antibiotics) at a density of 5 × 10^3^ cells/cm^2^ (day 0). The next day, spent mediums were aspirated and standard chondrogenic medium supplied with Bmp4 (50 ng/mL) and dexamethasone (100 nM) was added (day 1). The standard chondrogenic medium supplied with Bmp4 and dexamethasone was refreshed every day for three days (day 1 to day 3). At day 4, some cells were collected for analysis while others were divided into 4 subgroups for further treatments. The chondrogenic cultures were collected at day 9 for the analysis of chondrogenic markers. 

### 4.4. Cell Proliferation and Cell Viability

The chondrogenic cultures were dissociated by trypsin-EDTA for 10 minutes at 37 °C and suspended in standard chondrogenic medium. The cell numbers were counted with trypan blue staining and Countess II (Invitrogen, MA, USA). The viable cell numbers and the cell viabilities were documented.

### 4.5. Cell Morphology

The morphologies of iPS-OSH colonies and chondrogenic cultures were documented with a phase contrast microscope for the contrast enhancement. Digital photographs were taken at a magnification of 100X.

### 4.6. Safranin O and Alcian Blue Staining

The expressions of proteoglycans and glycosaminoglycans were detected by safranin O staining. Briefly, the chondrogenic cultures were gently washed with PBS (phosphate buffered saline) three times and fixed by 0.1% glutaraldehyde for 20 minutes at room temperature. After fixation, the cells were rinsed three times with PBS, and stained with 0.1% safranin O solution (Sigma-Aldrich) for 5 minutes right after a quick rinse of 1% acetic acid. Alcian blue (Sigma-Aldrich) was applied to stain the mucosubstances. After fixation, the cells were stained with alcian blue pH 1.0 solution overnight. The stained cells were kept in PBS. Digital photographs were taken at a magnification of 100X.

### 4.7. Real-Time PCR

Total RNA samples of chondrogenic cultures were collected by Trizol reagent (Thermo Fisher Scientific, Rockford, IL, USA). The expressions of genes (i.e., Gapdh, Sox2, Nanog, Klf4, T, Col1a1, Col2a1 and Acan) were measured by using Fast SYBR™ Green Master Mix (Applied Biosystems, MA, USA) and StepOnePlus™ Real-Time PCR Environment (Applied Biosystems). Briefly, 1 μg of total RNA from each sample was subjected to reverse transcription-PCR with Maxima H Minus First Strand cDNA Synthesis Kit (Thermo Fisher Scientific) according to the manufacturer’s protocol with oligo(dT)_18_ primers in a final volume of 25 μL. The real-time PCR reactions were performed with standard PCR reaction protocol by the manufacturer. Gapdh was detected as the reference gene for normalizing the level of mRNA expression.

### 4.8. Statistics

Data from at least three independent experiments were presented as mean ± s.d. Individual statistical tests were mentioned in figure legends, with significance established at *p* < 0.05. All statistical analyses were conducted using Prism 5.01 software (GraphPad Software, CA, USA, www.graphpad.com).

## Figures and Tables

**Table 1:** Primer sets for real-time PCR analysis.

Gene	NCBI Reference Sequence	Forward Primer Sequence	Reverse Primer Sequence
Gapdh	NM_008084.3	AGGTCGGTGTGAACGGATTTG	TGTAGACCATGTAGTTGAGGTCA
Col1a1	NM_007742.4	GCTCCTCTTAGGGGCCACT	CCACGTCTCACCATTGGGG
Col2a1	NM_031163.3	GGGAATGTCCTCTGCGATGAC	GAAGGGGATCTCGGGGTTG
Col10a1	NM_009925.4	TTCTGCTGCTAATGTTCTTGACC	GGGATGAAGTATTGTGTCTTGGG
Acan	NM_007424.2	CCTGCTACTTCATCGACCCC	AGATGCTGTTGACTCGAACCT
T	NM_009309.2	GCTTCAAGGAGCTAACTAACGAG	CCAGCAAGAAAGAGTACATGGC
Sox2	NM_011443.4	AGGGCTGGACTGCGAACTG	TTTGCACCCCTCCCAATTC
Sox9	NM_011448.4	AGTACCCGCATCTGCACAAC	ACGAAGGGTCTCTTCTCGCT
Nanog	NM_001289828.1	GAGCTATAAGCAGGTTAAGACC	TGCTGAGCCCTTCTGAA
Klf4	NM_010637.3	CCTTTCAGTGCCAGAAGT	ACTACGTGGGATTTAAAAGTGC

## Data Availability

All relevant data supporting the findings of this study is available within this Manuscript. Any further question or request should be made to the corresponding authors.

## Abbreviations

iPSC	Induced pluripotent stem cell
Tgfb1	transforming growth factor β1
Csf3	colony stimulating factor 3 (granulocyte)
Col1a1	Collagen Type I Alpha 1 Chain
Col2a1	Collagen Type II Alpha 1 Chain
Acan	Aggrecan
